# Prevalence and Molecular Characteristics of Avian-Origin *mcr*-1-Harboring *Escherichia coli* in Shandong Province, China

**DOI:** 10.3389/fmicb.2020.00255

**Published:** 2020-02-20

**Authors:** Xiaonan Zhao, Zhengjie Liu, Yin Zhang, Xiaomeng Yuan, Ming Hu, Yuqing Liu

**Affiliations:** ^1^Institute of Animal Science and Veterinary Medicine, Shandong Academy of Agricultural Sciences, Shandong, China; ^2^College of Life Sciences, Shandong Normal University, Shandong, China

**Keywords:** *Escherichia coli*, *mcr*-1, poultry, resistance, MLST

## Abstract

The objective of this study was to evaluate the prevalence and characteristics of avian-origin *mcr*-1-harbouring *Escherichia coli* in Shandong Province, China. During 2017—2018, a total of 668 non-duplicate *E. coli* isolates were separately collected from 8eight large intensive poultry farms in Shandong Province. Antimicrobial susceptibility testing for 10 antimicrobial agents commonly used in farms was performed on all *E. coli* isolates by the agar dilution method; the mobile colistin resistance gene (*mcr*-1) gene was screened by PCR, and *mcr*-1 positive isolates were PCR-screened for antimicrobial resistance genes and typed by multi-locus sequence typing (MLST). Among the 668 *E. coli*, 102 (15.3%) harbored the *mcr*-1 gene; high antimicrobial resistance rates were observed for ampicillin (100/102, 98.0%), followed by amoxicillin (99/102, 97.1%) and florfenicol (97/102, 95.1%), and a low level of resistance was found for amoxicillin/clavulanic acid (24/102, 23.5%). Five ESBL genes were detected, and all isolates carried *bla*_*TEM*_ (102/102, 100%), followed by *bla*_CTX–M_ (90/102, 88.2%). Four PMQR genes were detected; *aac(6)-Ib-cr* (40/102, 39.2%) was the most commonly isolated PMQR gene, followed by *qnrA* (10/102, 9.8%). Thirty-eight different kinds of STs were identified, and the dominant ST was ST93 (19/102, 18.6%), followed by ST48 (9/102, 8.8%). In summary, *E. coli* from poultry in Shandong could be a reservoir for the *mcr*-1 gene, which could pose serious risks to human public health.

## Introduction

Antimicrobial resistance is an ongoing severe public and animal health problem (Poirel et al., 2011). This issue occurs not only in pathogenic bacteria but also in non-pathogenic bacteria. The emergence of multidrug-resistant bacteria (MDR) has increased in the last decade and has caused public concerns around the world (Huang et al., 2018). Many studies have been reported that in Brazil, about 71.0% of the *E. coli* isolates from commercial chicken carcasses were MDR (Magiorakos et al., 2012); in Italy, 66.9% of the *E. coli* isolates from chicken meat were MDR (Ghodousi et al., 2015); in Egypt, 100% of the *E. coli* isolates from avian source were MDR (Enany et al., 2019), which demonstrated the high antimicrobial resistance. The addition of extensive subtherapeutic doses of antibiotics in animal feed is a very important reason for the rapid dissemination of antibiotic-resistant bacteria (Johnson et al., 2017).

Polymyxins are used to treat food-producing animals and have been considered the last resort antibiotics for the rapidly increasing MDR gram-negative pathogens, especially carbapenem-resistant *Enterobacteriaceae* (Pardon et al., 2012; Nation et al., 2015; Kaye et al., 2016). However, polymyxin use in animals and humans has promoted the selection and spread of *mcr*-1 drug-resistant strains (Sun et al., 2018). Since *mcr*-1 was discovered in food, animal and human isolates from Southern China, it has already spread to over 40 countries, implying that it played a prevalent role in the transferability of polymyxin resistance (Teo et al., 2017; Liu et al., 2016). In addition, *mcr*-1 positive plasmids could coexist with other resistance genes, notably ESBL genes; this has probably led to the emergence of pan-drug resistant strains and treatment failure (Haenni et al., 2016).

It has been reported that *mcr*-1 was restricted mainly to the Enterobacteriaceae, including *E. coli* (Olaitan et al., 2016). *E. coli* is a common inhabitant of the vertebrate intestinal tract and warm-blooded animals (Van den bogaard and Stobberingh, 2000). Moreover, avian-origin *E. coli* might hold zoonotic potential to cause human infections (Mellata et al., 2018).

China is one of the largest producers of chickens, especially in Shandong Province. In 2018, 1.65 billion commercial broilers were produced in Shandong Province, accounting for 41.2% of the country. An epidemiological survey of *E. coli* isolates from chickens from the early 1970s to 2014 suggested that the carriage rate of *mcr*-1 in China increased from 5.2% in 2009 to 30.0% in 2014 (Shen et al., 2016). However, in recent years, few studies have been performed on the prevalence and characteristics of avian-origin *mcr-1*-harboring *E. coli* in Shandong Province. Here, we aimed to determine the occurrence of the *mcr*-1-harboring *E. coli* isolated from poultry and their resistance profile between 2017 and 2018 in Shandong Province.

## Materials and Methods

### Ethics Statement

All procedures were approved by the Animal Care and Use Committee of Shandong Academy of Agricultural Sciences (SAAS-2019-021).

### Samples and Bacteria Isolation

From August 2017 to May 2018, 722 rectal swabs were collected from 8 large intensive poultry farms (farms 1-8) located in Weifang, Dezhou, Yantai, Qingdao, and Liaocheng regions in Shandong Province. The samples were independently collected from individual animals. The stock of poultry per farm was more than 200,000 heads. All the samples were transported in an ice box to our laboratory within 6 h for further analysis.

Each swab sample was inoculated in peptone water and cultured at 37°C for 24 h. A loop-full from incubated broth culture was streaked onto MacConkey’s agar and EMB plates. After incubation at 37°C for 24 h, suspected colonies were chosen by microscopical examination and further biochemical identification using the API 20E system (Sysmex bioMerieux, Tokyo, Japan). A total of 668 non-duplicate *E. coli* isolates were collected, and all of them were stored in Tryptic Soy broth plus glycerol (20%) at −20°C until further analysis (Table 1).

**TABLE 1:** The prevalence of *mcr*-1 positive *E. coli* isolates from poultry farms.

**Farms**	**Locations**	**No. of isolates**	**No. of *mcr*-1 positive isolates**
Farm 1	Weifang	62	4(6.5%)
Farm 2	Dezhou	72	5(6.9%)
Farm 3	Yantai	124	42(33.9%)
Farm 4	Qingdao	62	1(1.6%)
Farm 5	Qingdao	60	1(1.7%)
Farm 6	Yantai	102	42(41.2%)
Farm 7	Yantai	100	6(6.0%)
Farm 8	Liaocheng	86	1(1.2%)
Total		668	102(15.3%)

### *mcr*-1 Gene Detection

All *E. coli* isolates were grown in Tryptic Soy broth with shaking overnight at 37°C. According to the manufacturer’s protocol, genomic DNA was extracted with a TIANamp Bacteria DNA Kit (Tiangen, Beijing, China). All isolates were screened for the *mcr*-1 gene by polymerase chain reaction (PCR) using primers and conditions as previously described (Liu et al., 2016). The PCR amplicons were purified and subsequently sequenced (Sangon, Beijing, China), and the obtained DNA sequences were compared with those in GenBank using Basic Local Alignment Search Tool (BLAST).

### Antimicrobial Susceptibility Test

Antimicrobial susceptibility testing was performed on all *E. coli* isolates for 10 antimicrobial agents commonly used in farms including amoxicillin/clavulanic acid (AC), amoxicillin (AMO), ampicillin (AMP), ceftiofur (CEF), enrofloxacin (ENR), neomycin (NEO), doxycycline (DOX), florfenicol (FLO), gentamicin (GEN), and polymyxin (PB) by the agar dilution method recommended by the Clinical and Laboratory Standards Institute (CLSI, 2017); the susceptibility to colistin was interpreted according to the EUCAST guidelines^1^. The *E. coli* isolate ATCC 25922 was used as a quality control in this study.

### Detection of Antimicrobial Resistance Genes

*mcr*-1 positive isolates were characterized for extended-spectrum β-lactamase genes (ESBL) (*bla*_TEM_, *bla*_CTX–M_, *bla*_OXA_, *bla*_SHV_ and *bla*_PSE_) and plasmid-mediated quinolone resistance genes (PMQR) [*aac(6)-Ib-cr*, *qnrA*, *qnrB*, *qnrC*, *qnrD*, *qepA*, *oqxA*, and *oqxB*] by PCR using previously reported primers and conditions (Ahmed et al., 2013; Li et al., 2013). PCR products were visualized on 1.5% agarose gels, and the images were captured using image Capture Systems (Tanon-2500, Shanghai, China).

### MLST

The *mcr*-1 positive isolates were characterized using the MLST scheme. MLST analysis was carried out by sequencing fragments of seven housekeeping genes (*adk*, *fumC*, *gyrB*, *icd*, *mdh*, *purA*, and *recA*) as described online^2^. The PCR amplicons were purified and subsequently sequenced (Sangon, Beijing, China), and the alleles and sequence types (STs) were assigned according to the MLST database^3^. A minimum spanning tree was created using BioNumerics software 6.5 (Applied Maths, Kortrijk, Belgium) to analyze the distribution of STs.

### Statistical Analysis

Statistical comparisons of resistance to the 10 antimicrobial agents between *mcr*-1 positive isolates and *mcr*-1 negative isolates were analyzed using Fisher’s exact test or the chi-square test, which were performed using GraphPad INSTAT (GraphPad Software, Inc., United States). A value of *P* < 0.01 was considered significant.

## Results

### Prevalence of *mcr*-1 in Poultry Farms

Over the course of the study, 668 *E. coli* isolates were collected, and 102 (15.3%) of these were found to harbor the *mcr*-1 gene. The 102 *mcr*-1 positive isolates originated from poultry of farm 1 (*n* = 4), farm 2 (*n* = 5), farm 3 (*n* = 42), farm 4 (*n* = 1), farm 5 (*n* = 1), farm 6 (*n* = 42), farm 7 (*n* = 6), and farm 8 (*n* = 1). The prevalence of *mcr*-1 was 15.3% (102/668) (Table 1).

### Antimicrobial Susceptibility Test

Of the 102 *mcr*-1 positive isolates, resistance to AMP was common (100/102, 98.0%), followed by resistance to AMO (99/102, 97.1%) and FLO (97/102, 95.1%), and a low level of resistance was found for AC (24/102, 23.5%). In addition, the 102 *mcr*-1 positive isolates showed higher resistance to CEF, ENR, GEN and PB than the *mcr*-1 negative *E. coli* isolates (Table 2).

**TABLE 2:** The *mcr*-1 positive *E. coli* and the *mcr*-1 negative *E. coli* isolatesfrom poultry farms.

***Antimicrobials***		**No. of isolates**	
		
	***mcr*-1 positive isolates (n = 102)**	***mcr*-1 negative isolates (n = 566)**	**p-Value**
AC	24	90	0.08
AMO	99	506	0.02
AMP	100	509	0.02
CEF	86	233	<0.0001
ENR	81	326	<0.0001
NEO	74	337	0.02
DOX	91	527	0.45
FLO	97	548	0.56
GEN	66	165	<0.0001
PB	75	54	<0.0001

*Factors impacting mating in *P. infestans* Factors impacting mating in *P. infestans**

### Detection of Antimicrobial Resistance Genes

Among the 102 *mcr*-1 positive isolates, five ESBL genes were detected, all the isolates carried *bla*_TEM_ (102/102, 100%), followed by *bla*_CTX–M_ (90/102, 88.2%), *bla*_PSE_ (30/102, 29.4%), *bla*_OXA_ (16/102, 15.7%) and *bla*_SHV_ (4/102, 3.9%). Four PMQR genes were detected; *aac(6)-Ib-cr* (40/102, 39.2%) was the most commonly isolated PMQR gene, followed by *qnrA* (10/102, 9.8%), *qnrB* (4/102, 3.9%), and *qnrC* (1/102, 0.9%), and no other PMQR genes were detected (Table 3).

**TABLE 3:** Resistance phenotype, STs and resistance genes in the *mcr*-1 positive *E. coli* isolated from poultry farms.

**Farms**	**NO.**	**Resistance phenotype**	**ST**	**Resistance genes**
Farm 1	1-1	AC-AMO-AMP-CEF-DOX-ENR-FLO-GEN-NEO-PB	1251	*bla*_TEM_, *bla*_CTX–M_
	1-2	AC-AMO-AMP-CEF-DOX-ENR-FLO-GEN-NEO-PB	219	*bla*_*TEM*,_ *aac(6)-Ib-cr*
	1-3	AMO-AMP-CEF-DOX-ENR-FLO-GEN-PB	4710	*bla*_TEM_, *bla*_CTX–M_
	1-4	AMO-AMP-CEF-DOX-ENR-FLO-GEN-NEO-PB	10	*bla*_TEM_, *bla*_PSE,_ *aac(6)-Ib-cr*
Farm 2	2-1	AC-AMO-AMP-DOX-ENR-FLO-GEN	4477	*bla*_TEM_, *bla*_SHV_, *bla*_CTX–M_
	2-2	AC-AMO-AMP-DOX-FLO	4477	*bla*_TEM_, *bla*_PSE,_ *bla*_SHV_, *bla*_CTX–M_
	2-3	AC-AMO-DOX-NEO	4477	*bla*_TEM_, *bla*_CTX–M_
	2-4	AC-AMP-DOX-FLO	4477	*bla*_TEM_
	2-5	AC-AMP-DOX-ENR-FLO	4477	*bla*_*TEM*,_*aac(6)-Ib-cr*
Farm 3	3-1	AMO-AMP-CEF-DOX-FLO-GEN-NEO-PB	93	*bla*_TEM_, *bla*_PSE_, *bla*_CTX–M,_ *aac(6)-Ib-cr*
	3-2	AMO-AMP-CEF-DOX-ENR-FLO	93	*bla*_TEM_, *bla*_PSE_, *bla*_CTX–M,_ a*ac(6)-Ib-cr*
	3-3	AMO-AMP-CEF-DOX-ENR-FLO	93	*bla*_TEM_, *bla*_PSE_, *bla*_CTX–M,_ *aac(6)-Ib-cr*
	3-4	AMO-AMP-CEF-DOX-ENR -GEN-NEO-PB	93	*bla*_TEM_, *bla*_PSE,_ *aac(6)-Ib-cr*
	3-5	AMO-AMP-CEF-DOX-ENR-FLO	93	*bla*_TEM_, *bla*_PSE,_ *aac(6)-Ib-cr*
	3-6	AMO-AMP-CEF-DOX -FLO-GEN-NEO-PB	93	*bla*_TEM_, *bla*_PSE_, *bla*_CTX–M_
	3-7	AMO-AMP-CEF-DOX -FLO-GEN-NEO-PB	93	*bla*_TEM_, *bla*_PSE_, *bla*_CTX–M,_ *aac(6)-Ib-cr*
	3-8	AMO-AMP-CEF-DOX -FLO-GEN-NEO-PB	423	*bla*_TEM_, *bla*_CTX–M_
	3-9	AMO-AMP-CEF-DOX-GEN-NEO-PB	93	*bla*_TEM_, *bla*_PSE_, *bla*_CTX–M,_ *aac(6)-Ib-cr*
	3-10	AMO-AMP-CEF-DOX-ENR-FLO-GEN-NEO	93	*bla*_TEM_, *bla*_PSE_, *bla*_CTX–M,_ *aac(6)-Ib-cr*
	3-11	AMO-AMP-CEF-DOX-ENR-FLO-GEN-NEO-PB	10	*bla*_TEM_, *bla*_PSE_, *bla*_CTX–M_
	3-12	AMO-AMP-CEF-DOX-ENR-FLO	95	*bla*_*TEM*,_ *aac(6)-Ib-cr*
	3-13	AMO-AMP-CEF-DOX -FLO-GEN-NEO-PB	93	*bla*_TEM_, *bla*_PSE_, *bla*_CTX–M,_ *aac(6)-Ib-cr*
	3-14	AMO-AMP-CEF-DOX-ENR-FLO-PB	93	*bla*_*TEM*,_ *aac(6)-Ib-cr*
	3-15	AMO-AMP-CEF-DOX-ENR-FLO-GEN-NEO	93	*bla*_TEM_, *bla*_PSE_, *bla*_CTX–M,_ *aac(6)-Ib-cr*
	3-16	AMO-AMP-CEF-DOX-ENR-FLO	95	*bla*_TEM_, *bla*_PSE_, *bla*_CTX–M,_ *aac(6)-Ib-cr*
	3-17	AMO-AMP-CEF -ENR-FLO	224	*bla*_TEM_, *bla*_CTX–M_
	3-18	AC-AMO-AMP-CEF-DOX -FLO-GEN-NEO-PB	10	*bla*_TEM_, *bla*_CTX–M,_ *aac(6)-Ib-cr*
	3-19	AC-AMO-AMP-CEF-DOX-ENR-FLO-GEN-NEO	93	*bla*_TEM_, *bla*_PSE_, *bla*_CTX–M,_ *aac(6)-Ib-cr*
	3-20	AC-AMO-AMP-CEF-DOX -FLO-GEN-NEO-PB	10	*bla*_TEM_, *bla*_PSE_, *bla*_CTX–M_
	3-21	AMO-AMP-CEF-DOX-ENR-FLO-GEN-NEO	93	*bla*_TEM_, *bla*_PSE_, *bla*_CTX–M,_ *aac(6)-Ib-cr*
	3-22	AMO-AMP-GEN	10	*bla*_TEM_, *bla*_PSE_, *bla*_CTX–M_
	3-23	AMO-AMP -DOX-ENR-FLO-GEN-NEO	168	*bla*_TEM_, *bla*_PSE_, *bla*_CTX–M,_ *aac(6)-Ib-cr*
	3-24	AC-AMO-AMP-CEF-DOX-ENR-FLO	93	*bla*_TEM_, *bla*_PSE,_ *bla*_CTX–M,_ *aac(6)-Ib-cr*
	3-25	AMO-AMP-CEF-DOX-ENR-FLO-GEN-NEO	93	*bla*_TEM_, *bla*_PSE_, *bla*_CTX–M,_ *aac(6)-Ib-cr*
	3-26	AC-AMO-AMP-CEF-DOX-FLO-GEN-NEO-PB	93	*bla*_TEM_, *bla*_PSE_, *bla*_CTX–M,_ *aac(6)-Ib-cr*
	3-27	AMO-AMP-CEF-DOX-ENR-FLO-GEN-NEO	95	*bla*_TEM_, *bla*_PSE,_ *bla*_CTX–M,_ *aac(6)-Ib-cr*
	3-28	AMO-AMP-CEF-DOX-ENR-FLO-PB	93	*bla*_TEM_, *bla*_PSE_, *bla*_CTX–M,_ *aac(6)-Ib-cr*
	3-29	AMO-AMP-CEF-DOX -FLO -NEO	48	*bla*_TEM_, *bla*_PSE_, *bla*_CTX–M_
	3-30	AMO-AMP-CEF-DOX-ENR-FLO-PB	1011	*bla*_TEM_, *bla*_CTX–M,_*aac(6)-Ib-cr*
	3-31	AC-AMO-AMP-CEF-DOX-ENR-FLO-GEN-NEO-PB	48	*bla*_TEM_, *bla*_CTX–M_
	3-32	AMO-AMP-CEF-DOX-ENR-FLO-NEO-PB	69	*bla*_TEM_, *bla*_CTX–M_
	3-33	AC-AMO-AMP-CEF-DOX -FLO-GEN-NEO-PB	101	*bla*_TEM_, *bla*_CTX–M,_ *qnrC*
	3-34	AMO-AMP-CEF-DOX-ENR-FLO	38	*bla*_TEM_, *bla*_CTX–M,_ *aac(6)-Ib-cr*
	3-35	AC-AMO-AMP-CEF-DOX-ENR-FLO-GEN-NEO-PB	43	*bla*_TEM_, *bla*_CTX–M,_ *qnrB*
	3-36	AMO-AMP-CEF-DOX-ENR-FLO-NEO-PB	43	*bla*_TEM_, *bla*_PSE_, *bla*_CTX–M,_ *aac(6)-Ib-cr*
	3-37	AMO-AMP-CEF-DOX-ENR-FLO -NEO-PB	602	*bla*_TEM_, *bla*_CTX–M_
	3-38	AC-AMO-AMP-CEF-DOX-ENR-FLO-GEN -PB	93	*bla*_TEM_, *bla*_CTX–M_
	3-39	AMO-AMP-CEF-DOX-ENR-FLO-GEN-NEO	48	*bla*_TEM_, *bla*_CTX–M,_ *aac(6)-Ib-cr*
	3-40	AMO-AMP-CEF-DOX-ENR-FLO-GEN-NEO-PB	4969	*bla*_TEM_, *bla*_CTX–M_
	3-41	AC-AMO-AMP-CEF-DOX-ENR-FLO	206	*bla*_TEM_, *bla*_CTX–M_
	3-42	AMO-AMP-CEF-DOX-ENR-FLO-GEN-NEO-PB	7584	*bla*_TEM_, *bla*_CTX–M_
Farm 4	4-1	AMO-AMP-DOX -FLO-GEN-NEO	48	*bla*_TEM_
Farm 5	5-1	AMO-AMP-CEF-DOX-ENR-FLO-NEO-PB	359	*bla*_TEM_, *bla*_CTX–M_
Farm 6	6-1	AMO-AMP-DOX-ENR-FLO-NEO	4969	*bla*_TEM_
	6-2	AMO-AMP-CEF-DOX-ENR-FLO-GEN-NEO-PB	1011	*bla*_TEM_, *bla*_CTX–M,_ *qnrA*
	6-3	AC-AMO-AMP-CEF-DOX-ENR-FLO-GEN-NEO-PB	8900	*bla*_TEM_, *bla*_CTX–M_
	6-4	AMO-AMP-CEF-DOX-ENR-FLO-GEN-NEO-PB	156	*bla*_TEM_, *bla*_CTX–M,_ *aac(6)-Ib-cr*, *qnrA*
	6-5	AMO-AMP-CEF-DOX-FLO-GEN-NEO-PB	156	*bla*_TEM_, *bla*_CTX–M_
	6-6	AMO-AMP-CEF-DOX-FLO-GEN-NEO-PB	271	*bla*_TEM_, *bla*_PSE_, *bla*_CTX–M,_ *qnrA, qnrB*
	6-7	AMO-AMP-CEF-DOX-ENR-FLO-NEO-PB	359	*bla*_TEM_, *bla*_CTX–M_
	6-8	AMO-AMP-CEF-DOX-ENR-FLO-NEO-PB	1011	*bla*_TEM_, *bla*_CTX–M,_ *aac(6)-Ib-cr*, *qnrA*
	6-9	AMO-AMP-CEF-DOX-ENR-FLO-GEN-NEO-PB	1286	*bla*_TEM_, *bla*_CTX–M_
	6-10	AMO-AMP-CEF-DOX-ENR-FLO-NEO-PB	359	*bla*_TEM_, *bla*_CTX–M_
	6-11	AC-AMO-AMP-CEF-DOX-ENR-FLO-NEO-PB	271	*bla*_TEM_, *bla*_CTX–M,_ *aac(6)-Ib-cr*, *qnrA*, *qnrB*
	6-12	AMO-AMP-CEF-DOX-ENR-FLO-NEO-PB	359	*bla*_TEM_, *bla*_CTX–M_
	6-13	AMO-AMP-CEF-DOX-ENR-FLO-GEN-NEO-PB	1011	*bla*_TEM_, *bla*_CTX–M,_ *qnrA*
	6-14	AMO-AMP-CEF-DOX-ENR-FLO-GEN	359	*bla*_TEM_, *bla*_CTX–M_
	6-15	AMO-AMP-CEF-DOX-ENR-FLO-NEO-PB	359	*bla*_TEM_, *bla*_CTX–M_
	6-16	AMO-AMP-CEF-DOX-ENR-FLO-NEO-PB	101	*bla*_TEM_, *bla*_CTX–M_
	6-17	AMO-AMP-CEF-DOX-ENR-FLO-GEN-NEO-PB	359	*bla*_TEM_, *bla*_CTX–M,_ *qnrA*
	6-18	AMO-AMP-CEF-DOX-ENR-FLO-NEO-PB	1286	*bla*_TEM_, *bla*_CTX–M,_ *qnrB*
	6-19	AMO-AMP-CEF-ENR-FLO-GEN-NEO-PB	1011	*bla*_TEM_, *bla*_CTX–M,_ *aac(6)-Ib-cr*, *qnrA*
	6-20	AC-AMO-AMP-CEF -ENR-FLO-NEO-PB	4753	*bla*_TEM_, *bla*_CTX–M,_ *qnrA*
	6-21	AMO-AMP-CEF-DOX-ENR-FLO-GEN-NEO-PB	4204	*bla*_TEM_, *bla*_CTX–M_
	6-22	AMO-AMP-CEF-DOX-ENR-FLO-PB	354	*bla*_TEM_, *bla*_CTX–M_
	6-23	AMO-AMP-CEF-ENR-FLO-GEN-NEO-PB	1011	*bla*_TEM_, *bla*_CTX–M_
	6-24	AMO-AMP-CEF-FLO-GEN-NEO-PB	189	*bla*_TEM_, *bla*_CTX–M_
	6-25	AMO-AMP-CEF-DOX-ENR-FLO-GEN-NEO-PB	1266	*bla*_TEM_, *bla*_CTX–M,_*aac(6)-Ib-cr*
	6-26	AC-AMO-AMP-CEF-DOX-ENR-FLO-PB	48	*bla*_TEM_, *bla*_CTX–M,_ *aac(6)-Ib-cr*
	6-27	AMO-AMP-CEF-DOX-ENR-FLO-GEN-NEO-PB	101	*bla*_TEM_, *bla*_CTX–M,_ *bla*_*OXA*,_ *aac(6)-Ib-cr*
	6-28	AMO-AMP-CEF-DOX-ENR-FLO-GEN-NEO-PB	156	*bla*_TEM_, *bla*_PSE_, *bla*_CTX–M_
	6-29	AC-AMO-AMP-CEF-DOX-ENR-FLO-NEO-PB	1011	*bla*_TEM_, *bla*_PSE,_ *bla*_CTX–M,_ *aac(6)-Ib-cr*
	6-30	AMO-AMP-CEF-ENR-FLO-GEN-PB	48	*bla*_TEM_, *bla*_CTX–M_
	6-31	AMO-AMP-CEF-DOX-ENR-FLO-GEN-PB	189	*bla*_TEM_, *bla*_CTX–M_
	6-32	AMO-AMP-DOX-ENR-FLO-GEN-PB	2732	*bla*_TEM_
	6-33	AMO-AMP-CEF-DOX-ENR-FLO-GEN-NEO-PB	156	*bla*_TEM_
	6-34	AMO-AMP-CEF-DOX-ENR-FLO-GEN-NEO-PB	4129	*bla*_TEM_, *bla*_CTX–M_
	6-35	AC-AMO-AMP-DOX-ENR-FLO-GEN-NEO-PB	648	*bla*_TEM_, *bla*_CTX–M_
	6-36	AMO-AMP-CEF-DOX-ENR-FLO-GEN-NEO-PB	617	*bla*_TEM_, *bla*_CTX–M_
	6-37	AMO-AMP-CEF-DOX-ENR-FLO-GEN-NEO-PB	48	*bla*_TEM_, *bla*_CTX–M_
	6-38	AMO-AMP-DOX-ENR-FLO-GEN-NEO-PB	7108	*bla*_TEM_, *bla*_CTX–M_
	6-39	AMO-AMP-CEF-ENR-FLO-GEN-NEO-PB	189	*bla*_TEM_, *bla*_CTX–M,_ *aac(6)-Ib-cr*
	6-40	AMO-AMP-CEF-DOX-ENR-FLO-GEN-NEO-PB	7108	*bla*_TEM_, *bla*_CTX–M_
	6-41	AMO-AMP-DOX-FLO-GEN-NEO-PB	48	*bla*_TEM_
	6-42	AMO-AMP-CEF -FLO-GEN-NEO-PB	189	*bla*_TEM_, *bla*_CTX–M_
Farm 7	7-1	ENR-PB	224	*bla*_TEM_, *bla*_CTX–M_
	7-2	AMO-AMP-CEF-ENR-FLO-GEN-NEO-PB	224	*bla*_TEM_, *bla*_CTX–M_
	7-3	AMO-AMP-DOX-ENR-FLO-PB	2847	*bla*_TEM_, *bla*_CTX–M,_ *qnrA*
	7-4	AMO-AMP-CEF-DOX-ENR-FLO-GEN-NEO-PB	117	*bla*_TEM_, *bla*_CTX–M_
	7-5	AMO-AMP-CEF-DOX-ENR-FLO-GEN -PB	117	*bla*_*TEM*,_ *aac(6)-Ib-cr*
	7-6	AC-AMO-AMP-CEF-DOX-ENR-FLO-GEN-NEO-PB	297	*bla*_TEM_, *bla*_SHV_, *bla*_CTX–M,_ *aac(6)-Ib-cr*
Farm 8	8-1	AMO-AMP-DOX-ENR-FLO-GEN-NEO-PB	48	*bla*_*TEM*,_ *bla*_SHV_, *bla*_CTX–M_

### MLST

**FIGURE 1:**
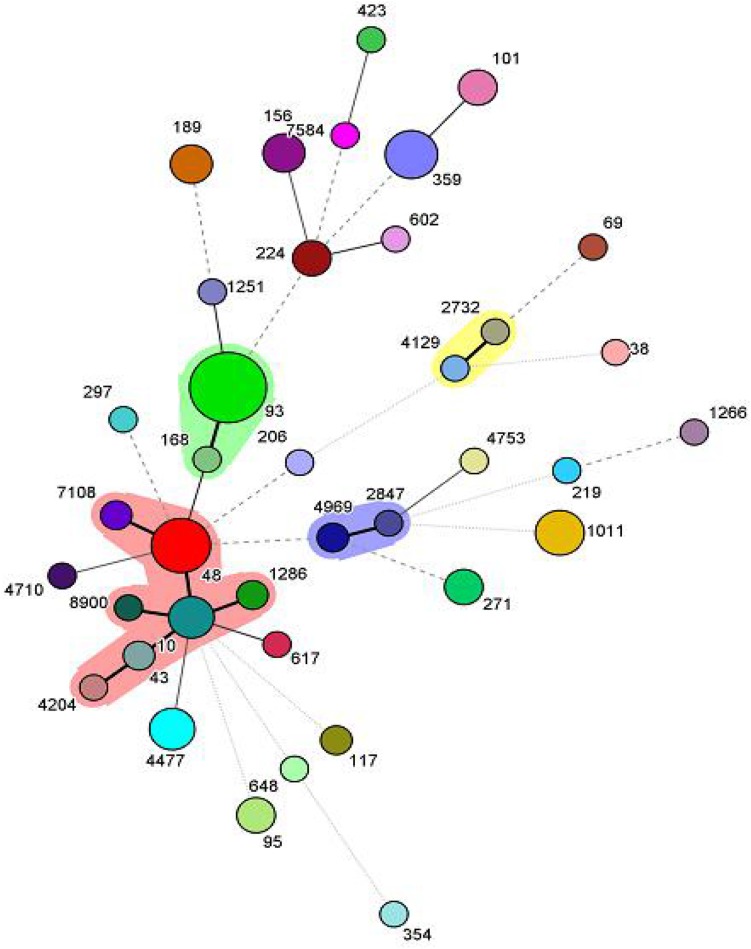
Minimum-spanning tree analysis of the 102 *mcr*-1 positive isolates. Each circle represents one ST, and the area of the circle corresponds to the number of isolates, the shadow region indicates that ST2732 and ST4129 belonged to one clone complex, ST2847 and ST4969 belonged to one clone complex, ST7108, ST48, ST1286, ST10, ST43, ST4204, and ST8900 belonged to one complex.

Among the 102 *mcr*-1 positive isolates, 38 different kinds of STs were identified. The dominant ST was ST93 (19/102, 18.6%), followed by ST48 (9/102, 8.8%), ST1011 (7/102, 6.9%) and ST359 (7/102, 6.9%). Of note, 73.7% (14/19) of ST93 isolates carried *bla*_TEM_, *bla*_PSE_, *bla*_CTX–M_, *aac(6)-Ib-cr*. BioNumerics software version 6.5 was used to generate a minimum-spanning tree based on all the sources of STs. As a result, ST2732 and ST4129 belonged to one clone complex, ST2847 and ST4969 belonged to one clone complex, and ST7108, ST48, ST1286, ST10, ST43, ST4204, and ST8900 belonged to one complex (Figure 1).

## Discussion

The colibacillosis of poultry caused by *E. coli* is the most common infectious disease worldwide (Mitchell et al., 2015; Liu et al., 2018). The growing selective pressure of antimicrobial treatments may contribute to the rapid spread of antimicrobial resistance genes (Guardabassi et al., 2004). In this study, we detected the *mcr*-1 gene in 102 (15.3%) *E. coli* isolates. The prevalence of *mcr*-1 (15.3%) in this study was higher than that in Japan; 9,303 *E. coli* were isolated from healthy animals between 2000 and 2014, and 39 (0.4%) of them were carried the *mcr*-1 gene (Kawanishi et al., 2017). In Europe, 10,206 *E. coli* were isolated from cattle, pigs and chickens between 2008 and 2014, 68 (0.7%) of which carried the *mcr*-1 gene (Farid et al., 2018). Such a high prevalence may be due to the selection of the farms tested and the methodology used. In addition, the different prevalence of *mcr*-1 in each farm might be associated with the different use of drugs and management in this study. Of note, in the same region, the prevalence of *mcr*-1 from farm 3 and farm 6 was higher than farm 7, this probably due to the different dosage of the polymyxin used in the farms.

In the current study, we noticed a high rate of resistance to AMP, AMO, and FLO in the *mcr*-1 positive isolates similar to a report in Eastern China from 2015 to 2017 (Zhuge et al., 2019), suggesting that these drugs may have been widely used in poultry farming. Most of the *mcr*-1 positive isolates showed susceptibility to AC, which was associated with the infrequent use of this drug, and the result was consistent with other reports (Dominguez et al., 2018). In addition, the *mcr*-1 positive isolates showed higher resistance to PB than the *mcr*-1 negative *E. coli* isolates in this study, which suggested that there was a close positive correlation between the resistance phenotypes and genotypes of the *mcr*-1 positive isolates. This result is consistent with other reports (Huang et al., 2017; Shen et al., 2018; Zhuge et al., 2019).

Extended-spectrum β-lactamase genes and *mcr*-1 phosphoethanolamine transferase enzymes have been identified as the main plasmid-mediated mechanisms of resistance to third-generation cephalosporins and colistin, respectively, and are currently considered a major concern both in human and veterinary medicine. Many studies of *E. coli* strains, most of which were isolated from animals, have demonstrated the presence of the *mcr*-1 gene together with ESBL genes (Rhouma and Letellier, 2017). In this study, all the *mcr*-1 positive isolates carried *bla*_TEM_, which was consistent with the result that 98.0% of the *mcr*-1 positive isolates were resistant to AMP. CTX-M-producing enterobacteria are widespread among human populations, but an increasing number of reports have described their presence in food animals and their environment (Li et al., 2007; Ewers et al., 2012; Lazarus et al., 2015). In the present study, 88.2% of the *mcr*-1 positive isolates carried *bla*_CTX–M_, which was similar to a previous study in which more than half of the chicken-origin *mcr*-1 positive strains presented the coexistence of the *mcr*-1 and *bla*_CTX–M_ genes (Yang et al., 2017), but higher than the 43.1% *mc*r-1 positive isolates carrying *bla*_CTX–M_ genes. The increasing prevalence and dissemination of co-carriage of ESBL genes in *mcr*-1 positive *E. coli* isolates found in our study suggested that it may add challenge to the global problem of difficult to treat and untreatable infections.

Quinolones are one of the main choices for the treatment of colibacillosis that occurs in humans and animals (Moawad et al., 2017). Thus, quinolone resistance is a public health issue that can lead to treatment failures and the use of alternative agents with greater side effects. In this study, *aac(6)-Ib-cr* was the most commonly isolated PMQR gene among the *mcr*-1 positive isolates, which was consistent with a previous report (Zhao et al., 2018). However, it was different from a report in Argentina that did not detect *aac(6)-Ib-cr* from *mcr*-1 positive isolates from poultry, and the most prevalent PMQR gene was *qnrB* (Dominguez et al., 2018). In addition, most isolates carried *aac(6)-Ib-cr*, which confer resistance to enrofloxacin and gentamicin. In this study, we also found the *qnrA*, *qnrB*, and *qnrC* genes. According to the results of this study, we suggest that *E. coli* from poultry could be a reservoir not only for the *mcr-1* gene but also for PMQR and ESBL genes.

In the current study, the MLST results revealed 38 distinct STs, which showed abundant diversity among *mcr*-1 positive isolates from different geographic locations. Notably, ST93 was the most common. It was different from that of previous studies that identified 40 distinct STs in 102 *mcr*-1 positive isolates, and ST48 was the most common (Yang et al., 2017). In addition, 73.7% of ST93 isolates carried *bla*_TEM_, *bla*_PSE_, *bla*_CTX–M_, *aac(6)-Ib-cr*, which were all from the same farm, indicating that the majority of the *mcr*-1 isolates from the same farm were probably clonal.

## Conclusion

Our results showed that the *mcr*-1 gene was present at a high prevalence in Shandong Province, and poultry can be an important reservoir of *mcr*-1 carrying *E. coli* strains. The wide range of phenotypic resistance to antibiotics in veterinary medicine was identified, and the high rate of antibiotic resistance gene-positive isolates detected suggest that defining avian-source *mcr*-1 positive isolates is critical for public health purposes.

## Data Availability Statement

The raw data supporting the conclusions of this article will be made available by the authors, without undue reservation, to any qualified researcher.

## Author Contributions

YL and XZ conceived and designed the study, wrote and revised the manuscript. YZ collected the samples. XZ, ZL, XY, and MH performed the experiments and analyzed the data.

## Conflict of Interest

The authors declare that the research was conducted in the absence of any commercial or financial relationships that could be construed as a potential conflict of interest.

## Footnotes

1 www.EUCAST.org

2 https://enterobase.readthedocs.io/en/latest/mlst/mlst-legacy-info-ecoli.html

3 http://enterobase.warwick.ac.uk/species/ecoli/allele_st_search
